# Clinical efficacy of electroacupuncture stimulation based on mismatch negativity in patients with prolonged disorders of consciousness

**DOI:** 10.1055/s-0046-1825520

**Published:** 2026-07-21

**Authors:** Xiaoguang Lang, Nuan Yang, Linxi Li, Zhengmao Xiang, Yuqin Zhao, Yuemin Gao, Zhixing Yao, Ying Lv, Sui Tian, Fang Hu, Xuehai Lv

**Affiliations:** 1 Handan Central Hospital, Department of Clinical Rehabilitation Medicine, Handan, Hebei, China.; 2 Hebei Medical University, Graduate School, Shijiazhuang, Hebei, China.; 3 Hebei North University, Graduate School, Zhangjiakou, Hebei, China.; 4 Chengde Medical University, Graduate School, Chengde, Hebei, China.

**Keywords:** Therapeutics, Acupuncture, Consciousness Disorders

## Abstract

**Background:**

The use of electroacupuncture can regulate brain activity, facilitating consciousness recovery.

**Objective:**

To investigate the clinical effectiveness of electroacupuncture at the Cuanzhu (BL2) and Sibai (ST2) accupoints on patients with prolonged disorders of consciousness (DOCs), and evaluate its awakening value.

**Methods:**

A total of 48 patients with prolonged DOCs were randomly divided into the control and electroacupuncture groups by the random number table method. The electroacupuncture group underwent treatment at the Cuanzhu (BL2) and Sibai acupoints (ST2) and was given electrical stimulation (2/100 Hz), while the control group received sham electroacupuncture stimulation for 3 weeks. Mismatch negativity (MMN) tests were administered to both groups before and after the intervention. Comprehensive assessments were performed using the Coma Recovery Scale-Revised (CRS-R) and the Glasgow Coma Scale (GCS) to compare the arousal rate and prognosis between the groups.

**Results:**

After treatment, both groups of patients showed a significant increase in MMN amplitudes and a reduction in latency ( *p* < 0.05). There was no statistically significant difference in MMN amplitudes and latency between groups after treatment. The awakening rate in the electroacupuncture group was 70.8%, and 54.2% in the control group. The difference between groups was not statistically significant ( *p* > 0.05).

**Conclusion:**

Electroacupuncture at Cuanzhu (BL2) and Sibai (ST2) may improve consciousness in patients with prolonged DOCs, but its therapeutic effect is not statistically superior to sham electroacupuncture. Due to the present study's small sample size and population heterogeneity, the findings should be interpreted cautiously.

## INTRODUCTION

In recent years, advancements in medical technology have led to the survival of patients with severe brain injuries following intensive treatments. However, these patients often experience varying degrees of sequelae, 1 notably disorders of consciousness (DOCs). A state where a patient remains unconscious for over 28 days is termed prolonged DOC (pDOC), encompassing conditions like the vegetative state (VS) and minimally-conscious state (MCS). 2 The etiology of pDOC remains unclear at present. Severe damage to the reticular activation system of the brainstem or central circuits can result in profound and enduring consciousness impairments. 3 The quality of life for individuals with pDOC is notably diminished, imposing a substantial burden on both families and society, and there is an urgent need for effective rehabilitation interventions.

Current treatment options for pDOC include hyperbaric oxygen therapy and neuroregulation techniques. 4 However, the former is costly and the latter lacks standardized stimulation parameters. In contrast, acupuncture, a traditional Chinese medical therapy, offers a cost-effective and straightforward alternative. Specifically, the Xingnao Kaiqiao acupuncture, 5 by stimulating the Cuanzhu and Sibai acupoints, brain activity can be regulated in a bottom-up manner, facilitating consciousness recovery. 5

The evaluation of therapeutic efficacy in patients with pDOC commonly relies on behavioral scales like the coma recovery scale – revised (CRS-R). However, the misdiagnosis rate is as high as 40%. 6 Mismatch negativity (MMN), a component of event-related potentials, serves as a neuroelectrophysiological marker linked to prefrontal attention processes in the brain. 7 Not influenced by active attention, MMN can be elicited during sedation, 8 anesthesia, 9 coma, 10 and similar states. There is a lack of strong evidence for electroacupuncture treatment of pDOC. Given the limitations of current treatment methods, we believe that electroacupuncture therapy is a potential, safe, and effective treatment method that deserves further exploration.

Patients with pDOC pose a significant clinical challenge, given the limited effective treatment options available. The high misdiagnosis rate of behavioral assessment scales and the drawbacks of existing therapies (e.g., high cost of hyperbaric oxygen therapy, lack of standardized parameters for neuroregulation techniques) highlight the urgent need for novel, evidence-based interventions. Electroacupuncture, as a traditional Chinese medical approach, has shown potential in neurorehabilitation, but its efficacy and mechanisms in pDOC remain unclear. This study aims to fill this gap by investigating the clinical effect of electroacupuncture at the Cuanzhu and Sibai acupoints on pDOC patients, using MMN as a key objective indicator, to provide new insights for clinical practice.

This study employed a randomized, single-blind, controlled experimental design to assess the clinical efficacy of electroacupuncture stimulation at the Cuanzhu and Sibai acupoints in patients with pDOC, focusing on indicators like MMN. The goal was to offer new evidence-based support for treating pDOC.

## METHODS

### Patients

Patients with pDOC in the Department of Rehabilitation Medicine of Handan Central Hospital were selected, between April 2024 and 2025. The present study was conducted in compliance with the principles of the Declaration of Helsinki. Approval for the study was obtained from the Ethics Committee of our hospital (June 18, 2024). Prior to participation, all patients provided informed consent and volunteered for the study. The ethics approval number for this study is Ethical Review Research No. 014, 2024. This randomized controlled trial has been registered on the International Standard Randomised Controlled Trial Number (ISRCTN, n. ISRCTN21011105) in accordance with the latest publishing requirements for randomized clinical trials.

#### *Inclusion criteria*

The inclusion criteria were fulfilling the requirements for a chronic DOC diagnosis. 11 Performing computed tomography (CT) or magnetic resonance imaging (MRI) scans to confirm presence of brain trauma, cerebral hemorrhage, cerebral infarction etc. Finally, having signed the informed consent form.

#### *Exclusion criteria*

The exclusion criteria were patients with known hearing impairment; history of severe cardiopulmonary diseases or other neurological disorders; metal implants that may interfere with EEG or electroacupuncture treatment; and presence of skin damage at the electrode placement site. Furthermore, those taking sedative-hypnotic drug intake during the MMN test. Those with incomplete clinical data or lost to follow-up were also excluded.

#### *Shedding standard*

Absence of both standard and deviant N1 during MMN detection in the patient. Cases in which the patient cannot tolerate the subsequent treatment.

### Experimental design

A single-center, single-blind, controlled design was employed. Patients were randomly allocated into two groups in a 1:1 ratio using the random number table method: the electroacupuncture group (n = 24, at Cuanzhu and Sibai acupoints, also receiving conventional treatment) and the control group (n = 24, conventional treatment along with sham electroacupuncture stimulation).

Sample size calculation was based on the primary outcome indicator of MMN amplitude change. Using G*Power (Heinrich Heine University) version 3.1, with an effect size f = 0.25, α = 0.05, and power (1-β) = 0.80, the calculated minimum sample size per group was 23. Considering a potential dropout rate of 10%, we initially planned to enroll 60 patients (30 per group). In general, the sample size for research is 5 to 10 times the number of items used in the scale. The CRS-R scale has 6 dimensions, so the sample size for this study should be between 30 and 60. As such, the sample of this study meets the requirements.

Drug therapy adhered to the guidelines outlined in the Chinese Expert Consensus on Rehabilitation of Neurocritical Illness (Part I). 12

### Data collection

All recruited patients' demographic data and laboratory results were gathered from the inpatient medical record system for the baseline evaluation. Prior to enrollment, MMN, GCS, and CRS-R tests were administered. At 3 weeks following enrollment and treatment, all patients had another MMN examination; and 3 months later, they all underwent follow-up. The Glasgow Outcome Scale (GOS) score was calculated during the follow-up.

Using the random number table approach, doctors who were not involved in the experiment independently assigned participants to the randomization, and none of the trial participants were given access to the pertinent grouping information. Rehabilitation physicians enrolled patients with pDoC, and the CRS-R and GCS scores were used for evaluation. The data gathering conditions were unknown to them. Professional acupuncture treatment technicians administer the electroacupuncture stimulation at the Cuanzhu and Sibai acupoints. Statisticians who were unfamiliar with the treatment allocation performed the data analysis.

### Intervention method

Rehabilitation nursing involved optimizing limb positioning and managing artificial airways. The techniques included joint range of motion exercises and breathing training.

**Figure 1:**
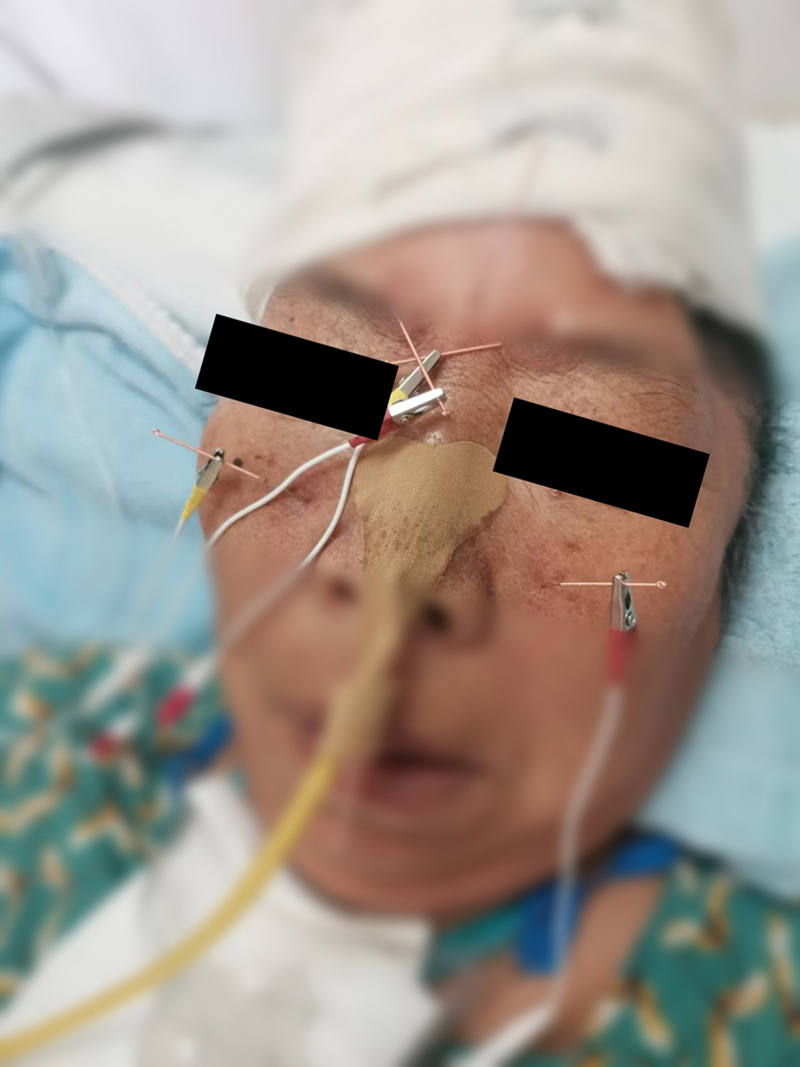
Stimulate the Zanzhu and Sibai acupoints with electroacupuncture.

Along with the appropriate acupoints of the V1 and V2 branches of the trigeminal nerve (i.e., the bilateral superior orbital foramen and inferior orbital foramen, with the corresponding acupoints on the body surface being Cuanzhu and Sibai, respectively), the electroacupuncture group received treatment using the Xingnao Kaiqiao acupuncture, 13 as shown in Figure 1 .

Bilateral Neiguan, Renzhong, and Sanyinjiao were chosen as the primary acupoints on the affected side, while Jiquan, Chize, and Weizhong were chosen as the secondary acupoints on the affected side for Xingnao Kaiqiao acupuncture. The selection of the Cuanzhu (BL 2) and Sibai (ST 2) acupoints is based on neuroanatomical and clinical evidence: these correspond to the exit sites of the ophthalmic (V1) and maxillary (V2) branches of the trigeminal nerve, respectively. Electrical stimulation at these points can specifically activate the trigeminal nerve branches, which project to the thalamus and prefrontal cortex via the trigeminal neural-thalamus-cortical pathway, thereby regulating brainstem reticular activation system and prefrontal lobe function related to consciousness arousal.

The electrical stimulation parameters (2/100 Hz sparse-dense wave, 1mA intensity) were determined based on previous studies on Xingnao Kaiqiao acupuncture and trigeminal nerve stimulation. 13 The 2/100 Hz frequency is commonly used in neurorehabilitation acupuncture to balance excitatory and inhibitory neural activities, and the 1mA intensity was set as the maximum tolerable dose for patients to ensure safety while maximizing therapeutic potential. Relevant studies have confirmed that this parameter combination can effectively increase cerebral blood flow and enhance neural cell survival rate. 14

### Procedure

The patient lies down in a supine posture. A 0.35 × 40mm disposable acupuncture needle of the Huatuo brand (Suzhou Medical Supplies Factory Co., LTD., Suzhou Medical Device Standard 20162200970) is chosen, following the standard disinfection of the acupoint skin with 75% ethanol. The electroacupuncture device is connected using sparse and dense waves at a frequency of 2/100 Hz, 1mA. The patient's tolerance should be the upper limit of the intensity. A 30 minute treatment should be held once a day, 5 days a week, for a duration of 3 weeks.

The Xingnao Kaiqiao acupuncture was used to treat the control group. They also received electroacupuncture differently. The control group, although connected to the electroacupuncture device, did not receive electricity. Patients did not actually receive electrical stimulation. All patients in the electroacupuncture group had the same course of treatment.

It is essential to keep a keen eye out for any negative responses that patients may have throughout therapy and respond to them promptly.

### Outcome

#### *MMN paradigm*

The Neuron-Spectrum-4/EPM digital neuroelectrophysiological equipment (Neurosoft LLC.) was used to identify MMN both before and after the treatment. The reference electrodes were positioned at the posterior mastoid processes of both ears, the grounding electrode was positioned at FPz, the recording electrodes were positioned at Cz and Fz, respectively, and the impedance between the electrodes was less than 5 kΩ in accordance with the International Electroencephalogram 10 to 20 system, which uses the Oddball mode. 14 There are 750 times as many stimuli in total. The analysis time is between −100 and 500 ms, and the amplifier bandwidth is between 0.1 and 30 Hz.

**Figure 2:**
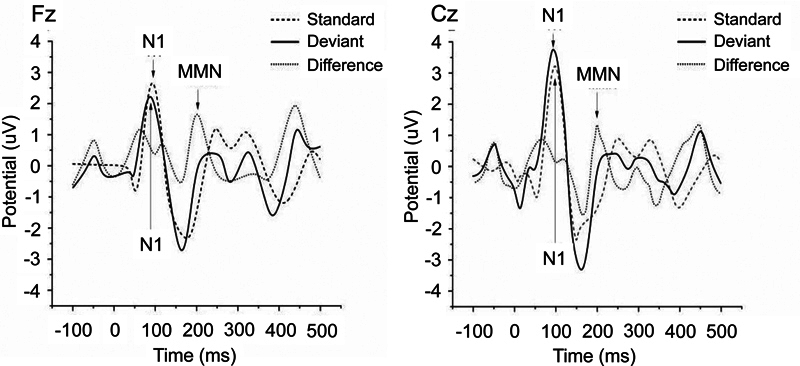
Mismatch negativity (MMN) waveform diagram.

The MMN, which stands for the waveform difference between the standard and deviation stimuli, 15 must meet the requirement that N1, the greatest negative wave that occurs within 100 to 300 ms, exists for both the standard and deviation stimuli. The amplitude must be noted and delayed at Fz and Cz. The MMN waveform schematic diagram is shown in Figure 2 .

#### *Clinical scale assessment*

##### CRS-R

Within 7 days before to treatment, two skilled neurologists performed five CRS-R assessments on the patients. The highest result determines the final CRS-R score. 16 The total score ranges from 0 to 23 points, with higher scores indicating better states of consciousness. The CRS-R scale includes six subdomains: auditory, visual, motor, oromotor/verbal, communication, and arousal. Each subdomain was scored separately, and the results are presented in detail in the Results section to provide a comprehensive assessment of consciousness recovery.

##### GCS

We recommend that two skilled neurologists evaluate each patient's GCS prior to treatment. 17 Lower scores indicate more severe consciousness disorders.

#### *Awakening rate*

Based on the Chinese Expert Consensus on the Diagnosis and Treatment of Prolonged Disorders of Consciousness 11 and relevant clinical criteria, 18 “awakening” in this study is defined as an improvement in the patient's consciousness level by at least one grade (e.g., from VS to MCS-, MCS- to MCS + , or MCS+ to EMCS), confirmed by both MMN changes (amplitude increase to the corresponding consciousness grade range) and CRS-R score elevation. This definition avoids the ambiguity of simply comparing consciousness status to admission and aligns with clinical practice for pDOC assessment. 11

The degree of consciousness is correlated with the amplitude of MMN. If the patient's MMN amplitude rises from the range of coma, vegetative state, MCS-state, and MCS+ state to the range that is one or more degrees of consciousness higher than the baseline level following a series of rehabilitation and awakening treatments, it suggests that the patient's degree of consciousness disorder has decreased in comparison to before. This article defines waking as the improvement of such patients' consciousness state, 18 and it calculates the awakening rate.

#### *Prognosis*

After 3 months from treatment conclusion, a follow-up will be carried out to assess the patients' prognosis. The GOS 19 will be used in structured telephone interviews 20 with family members or caregivers for the assessment. This scale classifies the outcomes of patients with craniocerebral injury into five categories (from death to good recovery, corresponding to 1–5 points respectively). 19

### Statistical methods

The IBM SPSS Statistics (IBM Corp.), version 27.0, was used to perform the statistical analysis. Frequency (n) was used to characterize counting data, whereas distribution features were used to pick statistical indicators for measuring data. The mean ± standard deviation (SD) was used to represent normally distributed data, while the median (IQR) was used to represent others. Data were classified using the Chi-squared test for statistical description of patient demographics, and data with a non-normal distribution were analyzed using the Mann-Whitney U test. A *p-* value < 0.05 represented a significant difference.

## RESULTS

### Comparison of general data between groups

**Figure 3:**
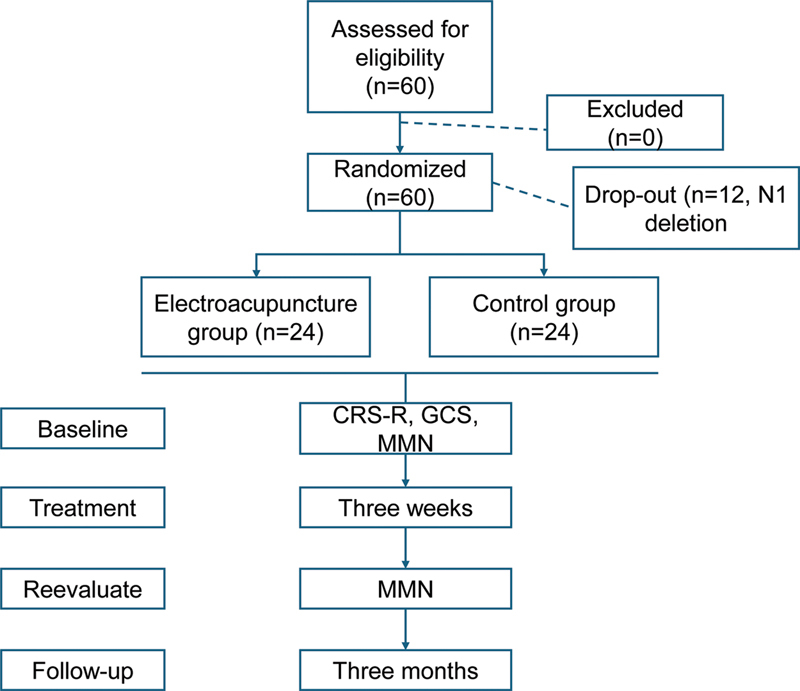
Participant enrollment flowchart.

The study flow diagram is displayed in Figure 3 . A total of 60 patients with chronic DOC admitted to the Department of Rehabilitation Medicine of the Handan Central Hospital from April 2024 to April 2025 were initially selected. The study subsequently excluded 12 patients due to the discovery of N1 deletion in MMN tests. The remaining 48 were randomly divided into two groups by the random number table method: the electroacupuncture group and the control group, with 24 cases in each group. No patients were lost to follow-up, and no adverse events were reported.

The two patient groups were equivalent and did not differ statistically significantly in terms of age, gender, duration of disease, GCS score, CRS-R score, consciousness level, or etiology ( *p* > 0.05), as shown in Table 1 .

**Table 1:** Comparison of general information between groups

		Electroacupuncture (n = 24)	Control (n = 24)	Z	*p* -value
**Age, years**		54.50 (50.50–66.75)	58.50 (53.2–64.00)	−0.557	0.557
**Duration, days**		41.50 (31.25–93.25)	46.50 (28.00–74.75)	−0.920	0.358
**GCS**		10.00 (9.25–12.00)	10.00 (9.00–11.00)	−0.780	0.435
**CRS-R**		13.50 (10.00–16.00)	11.00 (8.00–15.75)	−0.984	0.325
**Sex**	Male	20	18	0.505	0.724
	Female	4	6		
**Level of consciousness**	VS	2	4	1.221	0.791
	MCS-	11	12		
	MCS+	6	4		
	EMCS	5	4		
**Etiology**	Brain trauma	7	3	2.044	0.634
	Cerebral hemorrhage	16	20		
	Cerebral infarction	1	1		

Abbreviations: CRS-R, Coma Recovery Scale-Revised; GCS, Glasgow Coma Scale; EMCS, emergence from minimally conscious state; MCS, minimally conscious state; VS, vegetative state.

Note: Data are presented as numbers or median (IQR).

### Comparison of MMN amplitude and latency, CRS-R, and GCS before and after treatment

After 3 weeks of treatment, both groups demonstrated significant within-group improvements in MMN indicators (Fz/Cz amplitude elevation, latency reduction), CRS-R (total and subscores), and GCS score (all *p* < 0.05).

#### *MMN*

The electroacupuncture group showed greater amplitude increases (Fz: 1.37→2.23; Cz: 1.45→2.02; *p* < 0.01) and latency decreases (Fz: 182.00→139.00 ms; Cz: 176.50→145.50 ms; *p* < 0.01) when compared with the control group (Fz amplitude: 1.34→1.84; Cz amplitude: 1.51→1.69; *p* < 0.05; similar latency reductions, *p* < 0.01). No significant between-group differences were found in MMN parameters posttreatment (all *p* > 0.05).

#### *CRS-R*

The electroacupuncture group's total score increased from 13.5 to 18 ( *p* < 0.01) with all subscores improved, while the control group's total score rose from 11.0 to 16 ( *p* < 0.01) with corresponding subscore gains. Only the posttreatment total CRS-R score differed significantly between groups (Z = −1.978, *p* = 0.048), with no subscore differences (all *p* > 0.05).

#### *GCS*

**Figure 4:**
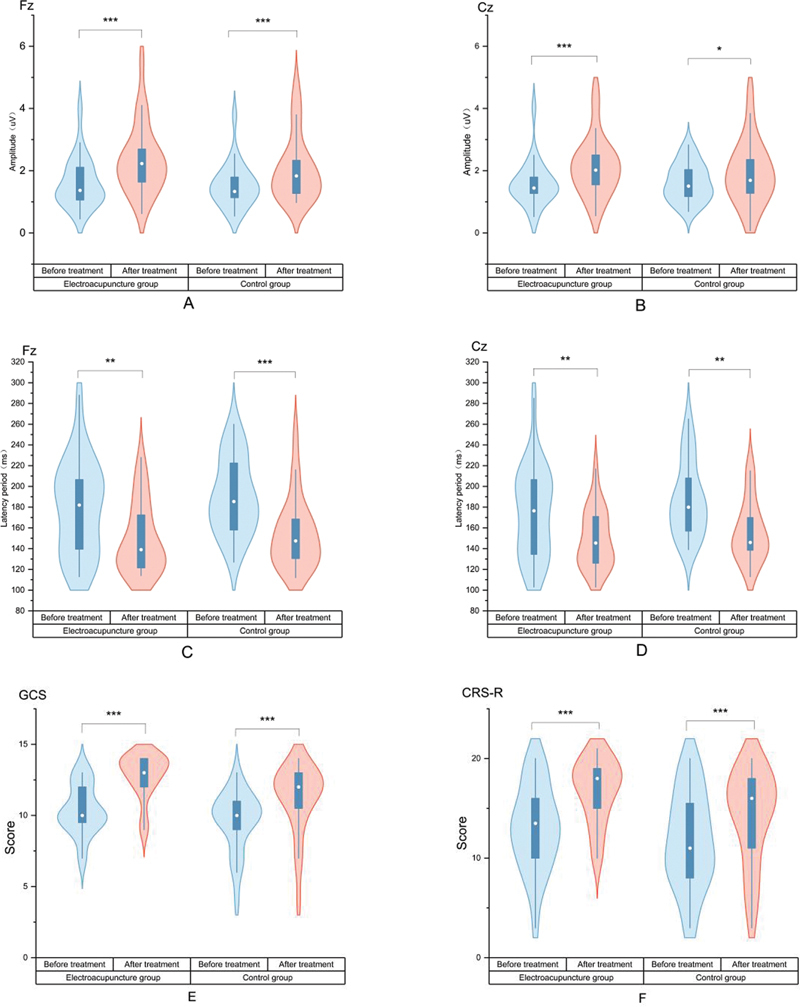
Comparison of mismatch negativity (MMN) data, coma recovery scale-revised (CRS-R), and Glasgow coma scale (GCS) before and after treatment between and within groups. Notes: A: Comparison of the Fz amplitude of MMN within the treatment group and the control group before and after treatment. B: Comparison of the Cz amplitude of MMN within the treatment group and the control group before and after treatment. C: Comparison of the Fz latency of MMN within the treatment group and the control group before and after treatment. D: Comparison of the Cz latency of MMN within the treatment group and the control group berore and after treatment. E?Comparison of the GCS within the treatment group and the control group berore and after treatment. F: Comparison of the CRS-R within the treatment group and the control group berore and after treatment.

The electroacupuncture group's score increased from 10.00 to 13 ( *p* < 0.01), and the control group's from 10.00 to 12 ( *p* < 0.01). The electroacupuncture group had a significantly higher posttreatment GCS score (Z = −3.050, *p* = 0.002). Data can be found in Tables 2 and 3 and Figure 4 .

**Table 2:** Comparison of MMN amplitude and latency, CRS-R, and GCS before and after treatment between groups

Group	N	Fz amplitude		Cz amplitude		Fz latency period		Cz latency period	
		Beforetreatment	Aftertreatment	Before treatment	After treatment	Before treatment	After treatment	Before treatment	After treatment
**Electroacupuncture**	24	1.37(1.05–2.12)	2.23(1.63–2.74) a	1.45(1.27–1.81)	2.02(1.55–2.52) a	182.00(137.7–207.25)	139.00(120.75–173.75) a	176.50(134.25–208.25)	145.50(125.50–171.50) a
**Control**	24	1.34(1.10–1.81)	1.84(1.24; 2.34) a	1.51(1.17–2.05)	1.69(1.24–2.39) b	185.50(158.00–225.25)	147.50(130.25–169.25) a	180.00(155.50–212.50)	146.00(138.25–170.00) a
**Z**		−0.062	−1.34	−0.443	−0.959	−0.877	−0.598	−0.980	−0.815
***p*** **-value**		0.951	0.180	0.658	0.338	0.381	0.550	0.327	0.415

Notes: Data are presented as numbers or median (IQR). ^a^ Compared with the indicators before treatment, *p-* value < 0.01. ^b^ Compared with before treatment, *p* < 0.05.

**Table 3:** Comparison of CRS-R (including subdomains) and GCS scores before and after treatment between groups

Indicators (scores)	Groups	N	Before treatment (median, IQR)	After treatment (median, IQR)	Z value	*p* -value
**CRS-R total**	Electroacupuncture	24	13.50 (10.00–16.00)	18.00 (14.50–19.00)	−4.219	< 0.001
	Control	24	11.00 (8.00, 15.75)	16.00 (10.50–18.00)	−3.987	< 0.001
	Comparison (after treatment)	–	–	–	−1.978	0.048
**CRS-R subdomain**	Electroacupuncture – Auditory	24	2.00 (1.00–3.00)	3.00 (2.00–4.00)	−3.872	< 0.001
	Control – Auditory	24	1.50 (1.00–2.00)	2.50 (2.00–3.00)	−3.640	< 0.001
	Electroacupuncture – Visual	24	2.50 (1.00–3.00)	3.50 (2.00–4.00)	−3.733	< 0.001
	Control – Visual	24	2.00 (1.00–3.00)	3.00 (2.00–4.00)	−3.464	0.001
	Electroacupuncture – Motor	24	3.00 (2.00–4.00)	4.00 (3.00–5.00)	−3.922	< 0.001
	Control – Motor	24	2.50 (2.00–3.00)	3.50 (3.00–4.00)	−3.801	< 0.001
	Electroacupuncture – Oromotor/Verbal	24	1.50 (1.00–2.00)	2.50 (2.00–3.00)	−3.686	< 0.001
	Control – Oromotor/Verbal	24	1.00 (0.00–2.00)	2.00 (1.00–3.00)	−3.420	0.001
	Electroacupuncture – Communication	24	1.00 (0.00–2.00)	2.00 (1.00–3.00)	−3.535	< 0.001
	Control – Communication	24	0.50 (0.00–1.00)	1.50 (1.00–2.00)	−3.290	0.001
	Electroacupuncture – Arousal	24	3.00 (2.00–4.00)	4.00 (3.00–5.00)	−3.964	< 0.001
	Control – Arousal	24	2.50 (2.00–3.00)	3.50 (3.00–4.00)	−3.774	< 0.001
**GCS**	Electroacupuncture	24	10.00 (9.25–12.00)	13.00 (12.00–14.00)	−4.327	< 0.001
	Control	24	10.00 (9.00–11.00)	12.00 (10.25–13.00)	−4.123	< 0.001
	Comparison (after treatment)	–	–	–	−3.050	0.002

Abbreviations: CRS-R, Coma Recovery Scale-Revised; GCS, Glasgow Coma Scale; IQR, interquartile range. Notes: Data were presented as numbers or median (IQR). The Mann-Whitney U test was used for comparisons within groups (before vs. after treatment) and between groups (after treatment); A *p-* value < 0.05 was considered statistically significant. CRS-R included six subdomains (auditory, visual, motor, oromotor/verbal, communication, and arousal) with a total score ranging from 0 to 23. Higher scores indicate better consciousness status. In GCS, lower scores indicate more severe consciousness disturbance.

### Comparison of the awakening rates of the two groups of patients

**Figure 5:**
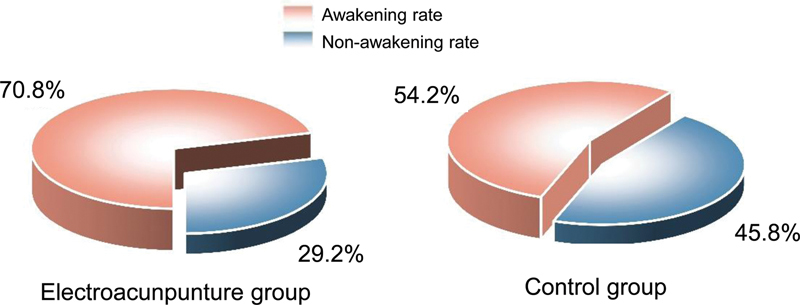
Comparison of the awakening rates of both groups.

According to the defined awakening criteria (consciousness level improved by at least one grade confirmed by MMN and CRS-R), 17 patients (70.8%) in the electroacupuncture group achieved awakening, compared with 13 (54.2%) in the control group. Although the rate of the electroacupuncture group was numerically higher, the difference between both groups was not statistically significant (χ ^2^ = 1.422; *p* = 0.233), as shown in Table 4 and Figure 5 .

**Table 4:** Comparison of the awakening rates of both groups

Group	N	Awake cases (N)	Awakening rate	Χ ^2^	*p* -value
**Electroacupuncture**	24	17	70.8%	1.422	0.233
**Control**	24	13	54.2%		

### Comparison of prognostic effects between the two groups of patients

**Figure 6:**
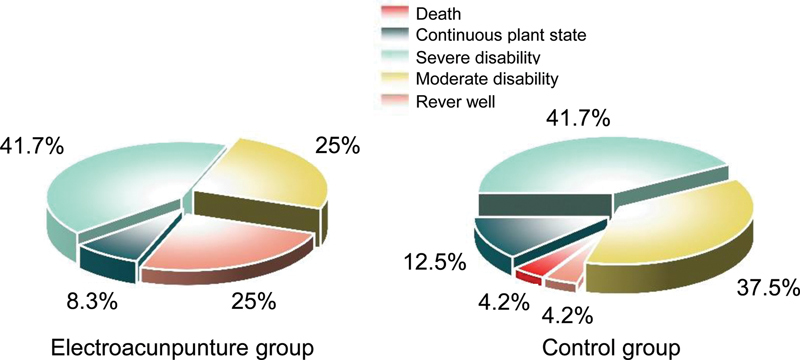
Comparison of prognostic effects between both groups.

At 3 months posttreatment, no statistically significant difference was observed in GOS grade distribution between the groups (Z = 5.228; *p* = 0.228), suggesting electroacupuncture had no superior long-term prognostic effect over sham procedure, as shown in Table 5 and Figure 6 .

**Table 5:** Comparison of the prognostic effects between groups

Group	N	Deaths	Continuous plant state	Severe disability	Moderate disability	Recovery	Z	*p* -value
**Electroacupuncture**	24	0	2	10	6	6	5.228	0.228
**Control**	24	1	3	10	9	1		

## DISCUSSION

The Xingnao Kaiqiao technique is one of the most popular electroacupuncture procedures. This study is based on neuroanatomy, clinical practice, and safety, selecting the Cuanzhu and Sibai points on the face. The two correspond to the exits of the ophthalmic (V1) and maxillary (V2) branches of the trigeminal nerve, and electrical acupuncture stimulation can equivalently activate the first and second branches of the trigeminal nerve. 21 The trigeminal neural-thalamus-cortical connection allows the stimulation to influence the prefrontal lobe and brainstem reticular structure. This offers patients with chronic problems of consciousness a novel approach to coma waking treatment.

According to studies, the locus coeruleus in the brainstem nuclei becomes more excitable when people awaken and their consciousness levels rise. 22 The trigeminal nerve either directly or indirectly projects the locus coeruleus. This provides the anatomical basis for electroacupuncture awakening.

Based on the Xingnao Kaiqiao acupuncture methods, the electroacupuncture group in this study incorporated electrical stimulation at the Cuanzhu and Sibai acupoints. In total, 3 weeks were allotted for treatment. Patients' level of consciousness waking was measured using MMN, a comparatively objective assessment instrument, both before and after treatment. The findings demonstrated that both the electroacupuncture and control groups showed significant within-group improvements in MMN amplitude, latency, CRS-R score, and GCS after 3 weeks of treatment ( *p* < 0.05). For example, the electroacupuncture group's CRS-R score increased from 13.5 (10.00–16.00) to 18 (14.5–19), and the control group's from 11.0 (8.00–15.75) to 16 (10.5–18).

However, there were no statistically significant differences in MMN amplitude, latency, awakening rate, or long-term GOS prognosis between groups ( *p* > 0.05). This result is consistent with Zheng's findings on the potential role of trigeminal nerve stimulation in consciousness regulation, though it does not support the existence of unique advantages of electroacupuncture over sham stimulation. 23 Additionally, some studies have pointed out that electroacupuncture stimulation of the trigeminal nerve can increase the oxygen content in brain tissue and enhance cell survival rate. 24 In order to awaken patients with chronic DOCs, electroacupuncture was chosen to activate the Cuanzhu and Sibai acupoints in this investigation, and certain therapeutic effects were observed.

The mechanism of electroacupuncture awakening can be explored from multiple dimensions, including neural electrical activity, metabolism, microcirculation, the integrity of neural circuits, and brain network functions. 25 Nevertheless, the study's findings demonstrated that, following treatment, there was no statistically significant change in MMN between the electroacupuncture and control groups.

One of the limitations of this study is that, while MMN can be used as an indicator to measure the clinical efficacy of patients with protracted DOCs, the multimodal integrated assessment of patients' clinical efficacy is more trustworthy. To increase the accuracy of the assessments, multimodal evaluations are therefore being considered for future research, 2 combining functional magnetic resonance imaging (fMRI) network analysis, CRS-R behavior assessment, and others.

Numerous factors affect the ability of patients with protracted DOCs to regain consciousness. 26 Given the heterogeneity of the sample in this study (including different etiologies such as brain trauma, cerebral hemorrhage, and cerebral infarction, as well as varying brain lesion locations), it is necessary to consider the potential correlation between treatment outcomes and these factors. For patients with cerebral hemorrhage, the location of hematoma may affect the sensitivity to trigeminal nerve stimulation—lesions involving the thalamus or prefrontal cortex may weaken the transmission of electroacupuncture signals through the trigeminal neural-thalamus-cortical pathway. In contrast, patients with brain trauma often have diffuse brain damage, which may lead to inconsistent responses to stimulation compared with those with focal lesions, like cerebral infarction. Future studies should stratify analyses by lesion location and etiology to clarify the populations that may benefit more from this therapy.

A notable limitation of this study is the higher dropout rate (20%), as 12 out of 60 initially enrolled patients were excluded due to N1 deletion in MMN tests. This may have reduced the statistical power of the study and potentially affected the results. The high dropout rate is mainly due to the strict inclusion criteria for MMN detection (requiring both standard and deviant N1 waves), which is a common challenge in studies using this test as an outcome indicator for pDOC patients. Future studies could optimize the MMN detection protocol or expand the initial sample size to compensate for potential dropouts.

In general, it is thought to be strongly associated with variables including age, the cause, and the progression of the illness. In contrast to Huang et al.'s study, 27 the majority of patients in the electroacupuncture (70.8%) and control (54.2%) groups had an improvement in consciousness; nevertheless, the difference in consciousness improvement between the groups was not statistically significant. One argument could be that there is not enough statistical power, since the included sample size is too small. Because of the sample size limitation, statistical significance cannot be achieved even if clinical differences are identified. Patients with extended disturbances of consciousness induced by electroacupuncture at Cuanzhu and Sibai acupoints did not have a statistically significant different prognosis from the control group. Severe brain injury, inadequate family care, and new issues in people with chronic DOC may be linked to this. Thus, it is critical to improve patient prognosis, decrease impairment, and enhance family care education for patients with chronic DOC.

In conclussion, by stimulating the trigeminal neural-thalamus-cortical connection, electroacupuncture stimulation of the Cuanzhu and Sibai acupoints may help patients with chronic DOC regain consciousness to some degree. This study does, however, nonetheless have certain shortcomings: The statistical power and results of this study were impacted by the small sample size and some differences in the etiology and degree of injury of patients with pDOC. As a result, the difference between the two groups did not reach statistical significance. To elucidate the effectiveness of electrotherapy for particular populations, future research must involve a larger patient population and perform group analysis according to the etiology, damage site, and degree of consciousness disturbance. The accuracy of DOC assessment should be increased when combined with multimodal assessment techniques like fMRI and positron emission tomography-computed tomography (PET-CT), in order to investigate the most effective awakening therapy plan for patients with chronic DOCs.
